# What the COVID-19 pandemic reveals about science, policy and society

**DOI:** 10.1098/rsfs.2021.0022

**Published:** 2021-10-12

**Authors:** Philip Ball

**Affiliations:** Freelance, London SE22, UK

**Keywords:** COVID-19, pandemics, policy, society, epidemiology

## Abstract

The global COVID-19 pandemic of 2020–2021 required politicians to work alongside and depend on scientists more closely than any other event in recent times. It also saw science unfold in real time under intense public scrutiny. As a result, it highlighted as never before the ways in which science interacts with policy-making and with society, showing with sometimes painful clarity that science does not operate in a social or political vacuum. With the advent of vaccines against the coronavirus that has caused the pandemic, science has come to be seen as something of a saviour. But at other times and in other contexts it has also been cast as a villain and an inconvenience, and has run into stark conflict with political leadership. In this article, I consider these issues with particular reference to the situation in the UK—which, as with any nation, illustrated some considerations of more general applicability but also had aspects unique to this country. I argue that there are many lessons to be learnt, and that, as this is surely not the last infectious-disease crisis of such magnitude that the world will face, we must hope they will be heeded.

## 1. Introduction

Pandemics and epidemics of infectious disease provide an unusually, even uniquely clear and stark picture of how science and society interact. While the lack of effective treatments or a detailed understanding of epidemiology severely limited possible scientific interventions in the Spanish flu outbreak of 1918–1919, the AIDS pandemic of the late 1980s and 1990s raised many of the issues that have been prevalent also during the COVID-19 pandemic. There too we witnessed an intensive and high-profile effort to understand the origin of that viral disease and to find treatments—resulting in Nobel prizes but also intense and often rancorous disputes between experts. The emerging scientific understanding of HIV led to calls for behavioural change, and motivated public information campaigns that highlighted the importance of clarity and frankness in communications about risk. There was an explosion of pseudoscientific ideas and conspiracy theories surrounding the disease, some of it abetted by the media and by prominent public figures (and some of which still persists today). Some sectors of society were hit harder than others, and the global effects of the outbreak were extremely heterogeneous. In the longer term, AIDS proved to have severe economic consequences for some countries.

AIDS-related illness has killed almost 33 million people worldwide, but there is now good reason to hope that the number of people who will die from COVID-19 will be a fraction of that. The pandemic caused by the SARS-CoV-2 coronavirus has, however, been far more disruptive to societies and economies, since the virus is transmitted more readily through everyday contacts and so societies have had to be put into the suspended animation of lockdowns to prevent this. As a result, the COVID-19 pandemic has held an even more powerful lens to the ways in which science and society interact.

The virus arrived at a particular febrile time for international affairs. Tensions between China (where the virus originated) and the USA were particularly high, and several countries, including the USA, UK, Brazil, India and Hungary, were governed by populist politicians of a libertarian or authoritarian character, elected in part because of deep social discontent. The USA was facing a fractious election and the UK was poised to withdraw from the European Union. As historian Margaret MacMillan has said, the pandemic has ‘brought into sharp relief flaws that were already starting to emerge in our globalized world: growing social and economic inequalities, for example, or the dangerous fragility of international supply lines' [1, p. 42].

At the time of writing, the COVID-19 pandemic is far from over; indeed, the appearance of at least one new fast-spreading variant of the virus has intensified the dangers and led to an even more lethal second wave of spreading in many countries, while others are experiencing a third. The development and approval of vaccines in record time has transformed the global outlook, but has not and will not solve the crisis in itself. In some ways, vaccines have added new complexity, for example by exacerbating the problems of inequality between nations, posing difficult regulatory decisions (such as whether to vaccinate children), creating a new source of denialism and pseudoscience, and heightening the selective pressures for the emergence of more challenging variants. The future still looks uncertain, and any meaningful end to the pandemic remains a distant prospect.

## 2. The UK as a case study

In understanding how science and society have interacted during the pandemic, more or less every nation has its own story. A global synthesis of them all is urgently needed, but beyond the scope of this article. I shall look predominantly at the case history with which I am most familiar—that in the UK—but will attempt to identify broader lessons where possible.

The political situation in the UK at the time that SARS-CoV-2 emerged was central to what followed. Four years of bitter political and social turmoil after the 2016 referendum on exiting the European Union led in December 2019 to the election of the Conservative prime minister Boris Johnson, with a large parliamentary majority. Johnson had succeeded the former Conservative leader Theresa May in July 2019, after May had been unable to secure a ‘Brexit’ deal that the British parliament would approve. Johnson, an avowedly populist leader who had expelled from the Conservative party those Members of Parliament who would not support his position on Brexit, began 2020 focused on his electoral promise to ‘Get Brexit done’. The country, however, remained highly divided on the matter, and the deal agreed at the eleventh hour with the EU was already creating economic problems and tensions in Northern Ireland, where the problem of border control (the EU–UK border there nominally being located along the historically fractious border with the Republic of Ireland) was still unresolved.

The UK's pandemic strategy initially diverged from that recommended by the World Health Organization and many epidemiologists and other health specialists worldwide. Rather than impose a lockdown and implement large-scale testing, it accepted the inevitability of the virus spreading through society and aimed to introduce measures that would merely slow that spread, so as to prevent the National Health Service from being overwhelmed, until herd immunity by natural infection was achieved. Only in mid-March 2020, after forecasts of perhaps hundreds of thousands of deaths in modelling studies of this ‘mitigation’ strategy, did the government change course and implement a national lockdown, which began on the 23rd.

A year later, the UK had suffered one of the worst mortality rates in the world: about 186 deaths per 100 000 of the population. The reasons for this are still debated. The UK fits with the linear correlation observed globally between COVID-19 mortality and obesity [2], the latter being a significant risk factor for respiratory diseases: the UK has the fourth highest prevalence of overweight people in the world. The country is also a hub of global connectivity, bringing many people carrying the virus into the country in the early days of the pandemic. However, there is widespread agreement among experts that the casualties and deaths cannot be explained by demographics alone, but resulted from the inadequacy of the response. Epidemiological modeller Neil Ferguson told *New Scientist* in June 2020 that ‘Part of [the reason] is clearly down to implementing lockdown relatively later than other countries did’ [3]. Professor of public health Helen Ward added that ‘We have been playing catch-up from the start’, citing the slow official response on testing, tracing and isolating people, and on social distancing.

While idiosyncratic in some respects, the UK was not dissimilar to many European countries in its initial response to the pandemic—which is to say, it seemed to watch almost paralysed as the inevitable threat approached. Despite horrific scenes of overwhelmed hospitals in Italy, the first European country to be hit by the virus, it took no effective early steps to combat the spread, and the British prime minister did not even attend the first meetings of the governmental body convened to deal with such a potential threat. International travel continued unchecked, several mass public gatherings went ahead and a national lockdown— recognized as inevitable by many scientists—did not happen until the virus had spread widely in the population. Ferguson has suggested [4] that twice as many people died in the first wave of infection as a result of the delay, relative to the likely outcome if the lockdown had come a week sooner.

This was the first of several tensions between what ‘the science’ seemed to recommend and the apparent constraints of other policy considerations. Commonly, the dilemma was framed in terms of the severe economic costs of lockdowns. These are undeniable: for example, essentially placing an economy ‘on ice’ raises the question of how wages will be paid, and decimates the income of many businesses, especially small shops, restaurants and other catering services that rely on passing trade. So the dilemma is a real one.

However, given that lockdowns were inevitable, it was always apparent that the best economic option was to introduce them sooner rather than later. There was never a supposed choice between ‘saving lives or saving the economy’: nations with the lowest mortality rates have tended to be those also that have suffered the lowest economic damage [5]. This is not the benefit of hindsight: some economists argued from the outset that tough measures to control the virus would allow for a faster and more vigorous reopening of the economy afterwards [6]. This is one instance where it appears that, in some countries, pandemic policy was formulated more on the basis of intuition than sound, cross-disciplinary advice.

Given the UK's strong scientific record in understanding infectious disease, and the fact that the Chief Medical Officer (CMO) Chris Whitty is himself a distinguished epidemiologist, it is puzzling why the country did not fare much better. Unlike the USA, where leading scientists were often conspicuously ignored or denigrated by President Donald Trump and his administration, in the UK there was a recognition from the outset that scientific advice would be crucial, and a well-established mechanism existed for feeding it into policy.

That is done in particular through the positions of the CMO and government chief scientific adviser (GCSA), currently Patrick Vallance, who has many years of experience in the pharmaceuticals industry. For emergencies like this, there also exists a tried and tested machinery of advice centred on the Scientific Advisory Group for Emergencies (Sage): a panel of leading experts that supplies advice to the Cabinet Office Briefing Room (COBRA) meetings and to other government departments. This system functioned well during the foot-and-mouth pandemic of 2001 (before Sage itself was formally established), producing a rapid and effective response. While this system serves the UK government as a whole, the devolved nations in Scotland, Wales and Northern Ireland also have their own advisory structures and have been free to determine their own pandemic measures such as lockdown restrictions.

The CMO, GCSA and their deputies have worked consistently with the government throughout the current crisis, in a system that was never designed with such a prolonged and extreme situation in mind. But while an emergency of this enormity is sure to strain any system, in this case there have been some glaring inconsistencies between what the best scientific opinion recommended for public health and what the government did—with very damaging consequences.

In the early days of the pandemic, decisions about public-health measures were hampered by a lack of knowledge about how the virus is transmitted. At first, there was considerable emphasis on transmission via contaminated surfaces (so-called fomites); decades of experience with respiratory diseases such as influenza suggested that transmission happened primarily via larger, rapidly settling droplets that fall on surfaces or are transferred by people's hands, and that smaller aerosol particles (less than 5 µm in diameter), which remain airborne, played little role. Public health advice therefore focused on hand-washing (the virus, having a lipid coat, is vulnerable to disruption by surfactants) and avoidance of face-touching, as well as on social distancing. Only gradually did evidence emerge that the predominant mode of transmission was indeed likely to be airborne [7,8]: the virus is carried between people by aerosol particles expelled by coughing, speaking or even breathing. The issue was still being debated in the summer of 2020, at which point the World Health Organization (WHO) had accepted only that aerosol transmission could not be ruled out. The US Center for Disease Control did not acknowledge that transmission can be airborne until October [9].

While this might look like procrastination in retrospect, it illustrates how hard it is to formulate sound policy when the science is so uncertain. Although the consensus is now that SARS-CoV-2 is predominantly transmitted by airborne particles, there remains some discussion about how effective masks are at reducing it. Yet even at the outset there was already plenty of evidence that the common practice in East Asian countries of wearing a mask in public if one has a cold or flu has some slight but significant effect in reducing infections [10,11]. One argument was, therefore, that, as the cost—both financial and in terms of convenience—of wearing masks was so low, it made sense to err on the side of caution until more information was available.

But the WHO initially advised against masks and expressed doubt about airborne infection [12]. In the UK, deputy CMO Jenny Harries suggested that masks could even be detrimental [13], as they might encourage face-touching and could cause infection when touched to remove them. This was puzzling, as there seemed to be no evidence to support the idea. There is a strong possibility that mask-wearing was resisted in Western countries not because of any scientific evidence for or against but because it was felt to be ‘unnatural’ to the traditional way of life. Resistance to the recommendation of masks among the general public might also have been partly motivated, in the UK at least, by the need to prioritize healthcare workers and hospital staff while supplies of good-quality masks were very limited.

Arguably the cause for concern here is not so much that some scientific advice turned out to be flawed—which is surely inevitable in the face of such an unknown threat—but that it was seemingly formulated more on the basis of intuition than sound evidence, and offered with false confidence. The same might be said of the suggestion by the UK chief scientists that lockdown should not be implemented too hastily because people might not be able to sustain it for long—the idea of ‘behavioural fatigue’. And a particular notorious and costly example of mistaken scientific advice was the initial idea that the virus might be better handled by allowing it to spread among those least likely to suffer serious illness (essentially younger people without pre-existing health conditions) to enable herd immunity to develop, while shielding the vulnerable.

Neither of these ideas was based on good evidence or reasoning. ‘Behavioural fatigue’ was not a concept recognized by the scientists in the government advisory group on behavioural science (called SPI-B). Its origin has never been clear, but seems to have arisen outside the community with the relevant expertize, and illustrates how the advice feeding into policy was sometimes opaque. Meanwhile, the idea of controlling a lethal infectious disease by the gradual achievement of ‘herd immunity’ through natural infection (the term was hitherto reserved for the management of such diseases by immunization, where it refers to the communal resistance acquired when a high enough proportion of the population has been immunized) was unheard of, and greeted with astonishment and horror by some experts outside the UK.

Nonetheless, herd immunity unquestionably formed part of the UK government's initial strategy to tackle the pandemic, notwithstanding the denial by the health minister Matt Hancock [14]. The idea was that the spread of the disease would merely be slowed by mitigation measures, so that the National Health Service was not overwhelmed at any stage, while allowing a sufficient proportion of the population to eventually become infected so that ‘natural herd immunity’ could develop. While that happened, people more vulnerable to COVID-19—which was known by then to pose a greater risk of mortality to older people, and to exhibit as a mild affliction in most people—would be shielded from infection by isolation.

This was never a realistic plan, as many experts pointed out at the time. First, there was not any understanding in March 2020 about whether infection by the virus even caused full and long-lasting immunity against re-infection. And even with efforts to shield the most vulnerable members of society (especially elderly people), it was always clear from what was known about the mortality rates that infection of perhaps 60% of the population (the threshold estimated to be necessary for herd immunity by Vallance at that time) would incur a massive death toll. Furthermore, it was not clear who, beyond the known enhanced susceptibility of older people, the ‘vulnerable’ were—many younger people, with or without pre-existing health conditions, have died, and many more are suffering from ‘long COVID’: health complications that might afflict them for years.

In any event, modelling studies of mitigation strategies that targeted a controlled approach to ‘herd immunity’, released on 16 March [15], showed that such strategies might incur massive fatalities and would overload the NHS. As a result, the UK government belatedly changed its plan, introducing a national lockdown on 23 March. Yet there was no clear admission of the flaws in the initial approach. Instead, ministers—and some scientists—have continued to deny that herd immunity was ever part of the plan.

The idea of ‘herd immunity’ (via ‘controlled protection’) as a means to combat the virus is now the genie let out of the bottle, and the idea has taken on a life of its own. It has continually resurfaced as an alternative strategy to lockdowns, which undoubtedly cause great short-term damage to the economy, to education and to public health, especially mental health. It was advocated in the so-called Great Barrington Declaration, a statement released in early October 2020 that was authored and signed by scientists who maintained the minority position of herd immunity as a viable strategy. Supported by a rightwing US thinktank, the Declaration has helped to sustain a small but vocal minority in the British media that spread misinformation about lockdowns and the progress of infections.

There is little doubt now that a herd-immunity policy would fail in any event. In Manaus, capital of the Brazilian state of Amazonas, the coronavirus was allowed to spread almost without any mitigation through the population, by October 2020 infecting around 76% of the population [16]. Yet even then herd immunity had not developed. Instead, Manaus had suffered a per capita death toll twice that even of hard-hit Britain—and this despite having a relatively young demographic. Adjusting for the age demographic, infection of that extent in the UK would cause around 350 000 deaths. The Manaus outcome shows that ‘pursuing herd immunity through naturally acquired infection is not a strategy that can be considered’, public-health experts Devi Sridhar and Deepti Gurdasani conclude [17].

Masks, meanwhile, have become one of the most politicized of behavioural issues, especially in the USA. Because they are so visible, they became virtually a badge of allegiance: an indication either that one accepts or rejects the dire threat and the need for restrictions on behaviour. The American attitude quickly split along highly polarized political lines—some regarded them as an imposition of the state, which many on the political right reject. While there now seems no doubt that mask-wearing can play a part, even if minor, in slowing the spread of infection, it continues to be derided by many who oppose lockdowns and behavioural curbs of all sorts.

All this should give scientists pause before pronouncing confidently on matters that have not yet been settled with clear evidence. It will always be hard for scientists, faced with uncertainties, to be able to express provisional views without seeing them seized and turned into arguments for or against particular policies by others with vested interests. But the pandemic has heightened the vital importance of clear and careful communication, with an honest admission of what is not known.

## 3. The importance of transparency and trust

David King, former GCSA in 2000–2008, cites a concern about transparency as one of the reasons why he set up Independent Sage, the group of experts (including some who advised the official Sage) that offers independent pandemic advice to the media and (if it cares to listen) to the government. ‘I set up Independent Sage’, King writes [18], ‘because I was, frankly, worried that communication was not clear from [Sage]. We were not told of any of their processes of decision-making, nor did we see the current Chief Scientific Adviser or Chief Medical Officer being made available to be challenged by the media’. That was not an inevitable consequence of the nature of these positions. The Phillips Commission report into the bovine spongiform encephalopathy (BSE) crisis in cattle in the early 1990s, released just before King himself became GCSA for the Blair government, concluded^1^ that it was essential that the government chief scientists be able to go into the public domain with their advice. The public needed to hear that advice to know if what ministers and politicians claimed were true, the Phillips report said. ‘That became my mantra during my time in this role’, says King. But what the public saw during the COVID-19 pandemic, in contrast, was the chief scientists carefully chaperoned by government members in their daily press conferences—and perhaps appearing thereby to lend their credibility to policy decisions they did not condone.

The constraints under which the chief scientists operate became particularly apparent during the controversy about government adviser Dominic Cummings' breach of lockdown rules in May 2020, when he drove his family from London to Durham after his wife had caught COVID-19, and then later made a trip to a scenic location nearby. (Cummings has since admitted that the public explanation he offered for leaving London for Durham was misleading and incomplete.) When the chief scientists were asked about the matter by reporters during a press conference in June, the prime minister intervened to prevent them from answering. When the press persisted and they had no option but to reply, both the GCSA and the CMO declared that the matter was a political one in which they had no wish to get involved.

Whether this was a genuine belief or one advised by the government must remain as matter of conjecture. But it was certainly incorrect. Such a visible violation of rules that most people had respected for months at great personal cost—sometimes being unable even to attend the funerals of dead family members—led to a sense of grave injustice. Such a serious blow to the arguments for compliance made the matter a public-health issue, which demanded a clear statement from the chief scientists of what the rules were and whether they had been breached. Subsequent studies [19] have made it clear that public trust in government rules and restrictions was significantly eroded by the affair.

In a crisis of these proportions, the roles of governmental scientific advisers are immensely challenging. Their formal obligation as civil servants is to provide ministers with objective expert advice and to support the government while recognizing that it is for politicians, not scientists, to formulate policies. All that sounds good and proper in principle. But it contains no obvious mechanism for acknowledging disagreement. If the scientific advisers feel that policy decisions are in conflict with the public interest, where then do their duties lie: with the government or the public? That is not a hypothetical question; it became increasingly apparent in September and October 2020 that the UK government's position conflicted with the advice from scientists, who were advocating more extreme measures (such as a short ‘circuit-breaker’ lockdown) in the face of rapidly rising infection cases. It subsequently became clear that this second wave after the summer lull was being driven by new, more infectious variants—and the second wave was considerably more lethal than the first. But the chief scientists seemed compromised in their ability to express dissent, leaving the public with the confusing scenario of press briefings in which the scientists would accompany policy announcements with statements that seemed in conflict with them. There is a strong case for considering whether scientific advisers, in the UK and perhaps more globally, need to be given more independence and scope for frank speaking than is usual for civil servants. At root, the tension here is a fundamental one: between the political demand for a unified message and the scientific importance of debate and dissent.

**Figure 1.**
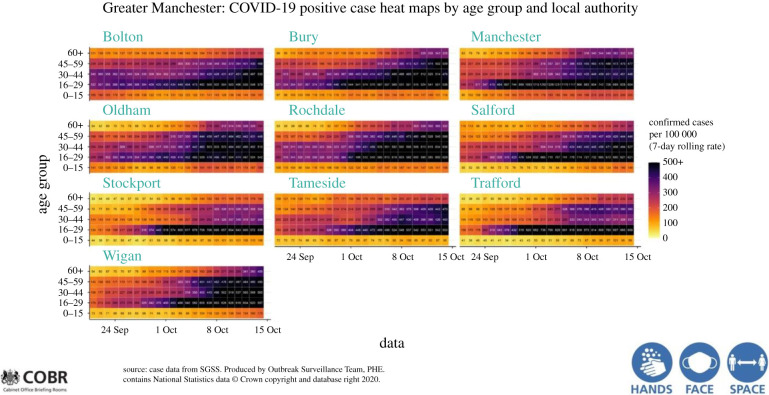
All clear? Overly detailed graphics at the governmental press briefings on the pandemic, often only partially and briefly visible on television screens, did not always help public understanding of the situation. Source: Outbreak Surveillance Team, Public Health England.

Another respect in which communications from the scientific advisers fell short during the crisis was in clarity. The GCSA, says King, ‘must be a good and clear communicator—language must be jargon-free, but be rigorous and accurate’. Whitty and Vallance were calm and sober in all their communications, and there is reason to believe that the public regarded them as trusted voices free of political spin, despite the evident constraints they were under. Throughout the pandemic, they were often seen as voices of stability and reason. But at times their contributions to the press conferences used convoluted graphics that were incomprehensible to the public both because of the complexity and the amount of information they contained (figure 1); even at a technical-scientific meeting they would have been challenging for the audience. Such shortcomings run the risk of making the scientific advice seem remote, out of touch and perhaps even an attempt to ‘blind us with facts’. Clear science communication is not a cosmetic, but an essential, part of science advice to policy and the public.

## 4. Politicized science

As the COVID-19 pandemic became a global threat in late February 2020, the British government stressed that its response would ‘follow the science’. Ministers repeated the phrase to the press like a mantra, apparently in the conviction that it would offer the public reassurance.

At the government's regular televised pandemic press briefings, a senior minister (often the prime minister Boris Johnson) would appear at a podium flanked by the chief scientific advisers. The politician would explain matters of policy, such as the announcement of changes to restrictions on behaviour and business, while the scientists would speak on the scientific data: the extent and demographics of infections, say, or progress with medicines. The notion was that the scientific information would explain and justify the policy decisions.

By the end of the year, the format for these briefings had not changed. But the phrase ‘following the science’ was no longer to be heard—because it was no longer credible. It was widely acknowledged that the government was making decisions that, while ‘listening to’ the science (in the new slogan coined around the late summer), were sometimes diverging from the recommendations of the scientific advisers. This did not in itself signify a breakdown in the relationship, but it clearly revealed a different kind of relationship to the one advertised at the outset. In the UK, at least, ‘following the science’ had been shown—for better or worse—not to be a viable way to govern.

That was no surprise to anyone familiar with the ways in which science and policy-making have long interacted. For them, the sight of a national leader flanked by his chief scientists rang immediate alarm bells. While on the one hand it might offer reassurance that politicians were taking expert advice, it blurred the boundaries between science and policy and raised questions about the independence of that advice. Not only is science—especially scientific understanding of such a new and imperfectly understood virus and its effects—never a monolithic and permanent body of knowledge, but scientific understanding neither can nor should in itself direct public policy and governance. It was inevitable and proper that political decisions had to admit a wider range of considerations, not least economic ones. Yet the non-scientific inputs to that process—cost–benefit analyses of particular policy choices, for example—were never made fully visible on the same basis, and in some cases it how appears that such considerations were never rigorously quantified or formulated. In other words, by presenting ministers alongside scientists, a misleading emphasis was being given to ‘the science’—making it all the easier for critics of policy to blame ‘bad’ scientific advice.

For a global crisis of this enormity, it was never going to be possible, or even desirable, to keep science and politics apart. That much has long been clear in efforts to address climate change. What was less obvious at the outset was just how quickly and alarmingly ‘the science’ would be politicized. The diversity of scientific viewpoints that is common whenever a new challenge arises, especially one so beset by unknowns and uncertainties, rapidly became a hunting ground for rival political ideologies. It was not difficult for all political players to find a scientific opinion that seemed to support their position, for example on how restrictive the measures should be that aimed to slow or arrest transmission of the virus. In some arenas, especially in the USA, opinions on the pandemic and the best response to it hardened into badges of tribal allegiance. It seems likely that the pandemic played a major part in determining the outcome of the US presidential election in November, in which Donald Trump, who held a strong position at the start of 2020 with the US economy looking fairly healthy, was defeated by the Democratic candidate Joe Biden. When it was announced on 2 October that Trump had tested positive for COVID-19, some parts of the media claimed (rightly or wrongly) that the entire electoral process was in jeopardy. After his recovery, Trump seemed less inclined than ever to find ways of mitigating the disastrous spread in the US, where at the time of writing the death toll stands at more than 600 000.

Meanwhile, what looked at first like a scientific question—how best to develop technologies and strategies for controlling and alleviating this lethal disease—soon proved to be at least equally a socioeconomic issue. Although national differences in the pandemic response initially not depended on GDP—many poorer nations initially fared better than some of the richest—within individual nations (notably so in the UK, for example) it has tended to be the most disadvantaged sectors of society that were the worst hit.^2^ The situation also highlighted social inequalities in other ways: people in better-paid desk jobs were able largely to continue their work from home, while more poorly paid manual workers suffered losses of earnings and the threat of unemployment. It was the lower-paid jobs—care workers, transportation drivers or delivery staff on zero-hours contracts—who proved to be the most indispensible, and who bore the brunt of the risks. The apparent differences in susceptibility to COVID-19 of different ethnic groups has added a racial aspect to these inequalities, although the reasons (and whether they are primarily socioeconomic) are still not fully understood^3^.

The pandemic has highlighted the shortcomings of international scientific and medical organizations. The WHO, for example, made some serious mistakes, such as in its initial advice on masks and transmission and its early reluctance to challenge the denialist narrative about the new virus issued by the Chinese state authorities. But the COVID-19 crisis has also illustrated the need for internationalism. Collaboration in the sharing of scientific information between nations has been essential, and the development of vaccines has been a truly global effort, helped by the existence of multi-national funding agencies and by international discussions on regulatory criteria [20]. International cooperation is likely also to be vital in ensuring that vaccines are made available rapidly to poorer nations. While the vaccine-sharing Covax programme^4^ is making efforts to facilitate this, there are also troubling signs that ‘vaccine nationalism’—attempts by individual nations to acquire the limited stocks of vaccines—may slow access in the developing world, perhaps even to the degree that some people might not get vaccinated before 2023.

Yet precisely because the rapid spread of the disease was a consequence of global inter-connectedness, the pandemic has shown that no nation can curtail transmission indefinitely until all have done so. At the same time, the necessity of controlling borders to slow transmission, as well as opportunistic misinformation that fuels xenophobia, has played into the nationalist sentiment of populist politics.

In such ways, the much vaunted internationalism of the scientific enterprise has sometimes collided with the tendency of political leaders to focus on national and even nationalistic narratives and priorities. In this respect as in others, the COVID-19 pandemic may carry some important lessons for efforts to slow and stop climate change, raising questions about just how much international treaties and discussions can realistically achieve. Lieven has argued that it might be more effective to mobilize national interests—a cause less alienating to the political right—to combat global heating [21]. Such arguments might be worth considering too for future preparations for pandemics (including the manufacturing and distribution capacity for vaccines), before assuming that only international actions can create more security.

## 5. Implications for science

Science has, since the start of the COVID-19 pandemic, never looked so important to the everyday lives of people everywhere. The characterization of the virus; development of tests for infection; information infrastructure to track the spreading; identification of new antiviral treatments and other drugs to reduce the severity of the disease; use of epidemiological models to predict the consequences of interventions; and the development of vaccines—all are dependent on scientific expertize and capability. All have benefitted from international collaboration and from the pre-existence of a strong scientific and technical research base.

On the other hand, one of the most striking outcomes of the pandemic is that how well a country has fared in avoiding mass illness and fatality is more or less entirely uncorrelated with either its wealth (as measured by, for example, per capita GDP) or its scientific strength. The USA and the UK have been among the countries worst hit, while many low-income countries in Africa and Asia initially fared well. (The spread of more infectious variants and the disparity of access to vaccines are now, however, changing that picture.) Philip Bobbitt, director of the Center for National Security at Columbia Law School, remarks on ‘the utter foolhardiness of the responses of countries, including those that might have been expected to do better precisely because they had the enormous state capacity, scientific expertize and educated populace to have fielded the state apparatus, social trust and leadership necessary to prevail in this sort of crisis' [22, p. 68]. While the reasons for these failures are complex, it is abundantly clear that scientific capacity or competence is of itself no shield against an event like this, if there is not the political aptitude or will to take effective action. Nations in which leaders acted swiftly and decisively—not hesitating to introduce significant constraints on public mobility, social distancing and border controls, and implementing effective regimes for testing, tracing and isolation—fared better than those that adopted a more laissez-faire approach. In general, there was no actual conflict between public health and economic outcomes: those countries that achieved low mortality rates have tended also to have suffered less economic damage, not least because they have been able to loosen lockdowns and other restrictions sooner so that life can resume.

Faced with such an immense, urgent and novel crisis, science has played out in real time in the public spotlight. In many ways, this may prove to be a good thing. How science works—with all the uncertainties and disagreements, the necessity of working with imperfect and incomplete information, and the need for cross-disciplinary dialogue and collaboration—has been displayed to public view as never before. Despite the insistence of some politicians in speaking of the ‘miracle’ of science, it has been shown to be anything but: it is the fruit of hard-won knowledge and diligent, careful research, stretching back many decades. (The mRNA vaccines, for example, were made possible by around two decades of prior research, which through good fortune reached fruition just in time to be useful.) But the inevitable uncertainties and disagreements have supplied footholds for fringe views, and some scientists have proved to be easily and even enthusiastically manipulated or exploited for other agendas.

What is more, it has never been more clear that the notion that modern societies are rationalistic and evidence-driven has always been a myth. The rash of pseudoscience and conspiracy theories about the nature, or even the reality, of the virus, the disease it causes, and the potential treatments and vaccines, is the equal of anything seen during the plague outbreaks of the Middle Ages, with the added problem that such ideas have instant global reach through the internet and social media. Little of this misinformation has appeared de novo or in isolation; rather, it has ridden on existing information highways created for political or other ends, such as extremist ‘hate’ networks, climate-change deniers or anti-vaccination groups [23]. While the WHO has dubbed this an ‘infodemic’^5^, the pandemic has shown that misinformation is already endemic in our societies, and that they are especially vulnerable to it in times of crisis. If there was already an urgent need to understand the epidemiology of misinformation because of its potential to fuel social and political unrest, we can now see what a threat it poses too to public health. The challenge is not simply one of controlling the technology—for example by social-media companies adopting and implementing anti-misinformation policies—but of understanding the underlying social psychology. For instance, at face value, the marked overlap between opposition to lockdowns and to vaccines (which might reasonably be regarded as the antidote to lockdowns) seems like the most extreme irrationality. But it surely stems from a deep-rooted antipathy to or fear of interventions perceived to constrain personal liberty, and will not be alleviated by ridicule or mandate.

The pandemic should (but might not) have dispelled any notion that science itself operates in an elevated sphere of pure reason separate from the social currents around it. That has never been true, but it is more obvious now than ever. At times, scientists seemed alarmingly siloed—as when, for example, modellers in the UK failed to factor in the realities of public health management and so overlooked the potential for infection to spread from the community into care homes. One of the constant criticisms of science advice in the UK was that it was insufficiently informed by public-health experts; that was one of the concerns motivating the creation of Independent Sage. And the impressive development of rapid tests for infection and facilities to analyse them counted for little in the face of logistical and infrastructural errors that undermined the ability to make effective use of the data. Beyond this, the ability of scientific interventions to make a difference were limited by the capacity of public healthcare—or, in the USA in particular among developed nations, by the disastrous lack of it for many sectors of society. The pandemic highlighted appalling social inequalities: socioeconomic status was, in wealthier nations, a key risk factor for becoming ill and dying from COVID-19. In short, good science and technology cannot compensate for failures of governance, management, or social justice. As experts in science and technology studies have long argued, there cannot be high-tech solutions to low-tech problems.

Finally, the pandemic has shown that it makes no sense to consider public health as anything other than a global issue. While nations became necessarily, and temporarily, more ‘isolationist’ with the closure of borders and strict immigration controls, it was always clear that no place will be indefinitely safe until everywhere is. Not only does that show the futility of national stockpiling of vaccines at the expense of the wider world, but it reveals the need for global efforts to identify, limit and control the inception and spread of disease in the first place. That issue has implications for urbanization and habitat protection, as well as for openness in the discourse between nations. In all these respects there are again lessons for dealing with climate change, which might itself fairly be posed as an urgent global public-health crisis with astronomical economic costs. Climate change, infectious disease, population increase, socioeconomic inequality and environmental degradation are all intimately connected; none can be solved in isolation, and all are highly contingent on the political climate.

## 6. The broad outlook: the value of trust

The diversity of pandemic outcomes and responses throughout the world makes it hard to draw any general conclusions from individual cases about how science, policy and society can and should interact. The interplay between scientific advice, public trust and adherence, and governmental policies has been varied and complicated. South Korea does not have a high degree of public trust in government or scientists, and yet there was good public compliance and effective coordination of resources, leading to one of the best responses to the pandemic. Sweden adopted a more relaxed strategy that avoided lockdowns—but although as a result it has suffered much higher fatalities than surrounding Scandinavian countries, to the extent that the strategy was declared a failure by the Swedish king, there seems also not to have been wide public discontent because the public were treated with trust and openness about the measures being adopted. The United States has seen perhaps the highest level of politicization of pandemic responses, with strong resistance to restrictions from libertarian leaders at the state and national level, and misinformation and denigration of scientific experts being encouraged even by the Trump government. The ideological divisions, and their lethal consequences, continue to be evident in the vaccination programme.

In the UK, it is not hard to imagine that the scientific advice might have led to a rather different strategic outcome under a government with less libertarian instincts, less inclined to adopt exceptionalist and nationalist narratives, and less reluctant to impose restrictions they feared would be unpopular. It seems clear that no mechanism for feeding science into policy will by itself prescribe the way in which that advice is understood and acted on, and one could argue that the advice was in this case insufficiently attuned to its intended audience. The careful, circumspect and sometimes even rather bland minutes of Sage meetings, for example, do not in retrospect seem likely to provoke the necessarily action leaders who were temperamentally disinclined to take it. Birch argues persuasively that extreme circumstances such as these justify scientific advisers offering “normatively heavy advice”, which includes specific policy recommendations that they would and should eschew in normal times [24]. This is all the more so when the character of policymakers is seen to be averse to the kinds of decisions advisers feel are warranted.

But while there were also some mistakes in the scientific advice itself—not least the initial acceptance of a strategy based on a perceived need to develop herd immunity through infection—on the whole the quality of the advice and the machinery for delivering it seem not to have exhibited the kind of deficiencies that can account for the UK's extremely poor performance in terms of health outcomes. Some of the biggest problems came from failures of implementation at the institutional and organization level—most notably, in creating a viable test–trace–isolate scheme. Contracts were awarded to private companies with little or no experience in public health that failed to deliver; logistical mistakes led to delayed or even mislaid test results; the testing system was overwhelmed at a vital stage in September 2020 due to an entirely predictable surge in demand; and there was too little economic provision to ensure that people could afford to self-isolate. The moral here is that technological knowhow, for which the UK does not by any means lack, is of little avail without the infrastructure to put it to use.

In assessing ‘what went wrong’, it would be a grave mistake to overlook the personal element in leadership, or lack thereof. A public inquiry into the handling of the pandemic has been promised by the UK government, but no timescale has been set for it. If and when it happens, it seems likely that much of the discussion will be focused on the factual basis for and the timeliness and appropriateness of decisions, the degree of preparedness for such an emergency, and so forth. Yet the full story looks far more Shakespearean. The government's chief adviser Dominic Cummings, now expelled from his role and locked in antagonism and recrimination with his former boss, has, in public testimony, painted an extraordinary picture of the prime minister as being callously unheeding of public suffering, at one point declaring that he would rather see bodies piling up in the streets in their thousands rather than initiate a second lockdown. Cummings himself has declared the former health secretary Matt Hancock to have been totally incompetent, and admits he was constantly battling to get Johnson to commit to decisions. Indeed, Cummings insists that he always regarded Johnson as unfit for the prime ministerial office, and says he was making plans ever since the December 2019 election to have him replaced. Hancock, meanwhile, has been forced to resign after being exposed in the media for having a physical relationship with a close colleague at a time that distancing rules made such an interaction illicit. That this level of dysfunction existed within the government as it was facing extraordinary difficult decisions on which millions of lives depended was surely as significant as any questions about the availability of PPE or corrupt awarding of pandemic-related contracts. And such deep flaws in the British political fabric are themselves symptomatic of the chaos and corrosion of public life that arrived in the wake of the Brexit referendum.

The personal rivalries and misdemeanours of ministers and their advisers might scarcely look like relevant fare in assessing the scientific aspects of how the pandemic was handled. But to neglect them would imbue the assessment with a false sense of normality. Similar stories can be told for, among others, the US, Italy, Brazil and India: the problems are in the end problems of governance and of democratic accountability. However unseemly, not to mention methodologically difficult, it might seem for a scientific assessment to take account of such factors, it would be woefully incomplete unless that is done. To confine the assessment to technical matters beggars the question.

At any rate, such an assessment is urgently needed—but in the face of the government's reluctance to launch an official inquiry, there seems to be little enthusiasm among British scientific bodies and institutions to take matters into their own hands by conducting their own reckoning. Some scientists have voiced the concern that such an inquiry would devolve into a blame game. But that need not and should not be so. There are many important questions such an inquiry should ask, not just about what science fed into policy but about the scientific, technological, industrial and public-health infrastructure that underpinned the pandemic response. To forgo such self-analysis (or relinquish it to political institutions) after what was arguably the biggest science-based crisis in living memory seems to this observer to be a profound failure of responsibility.

In part, it is a matter of establishing trust in science. For one of the lessons of the pandemic is that public trust is essential for managing a crisis of this sort. In the UK, ministers and other government advisers were seen to lack any accountability for their mistakes and misjudgements, and there was a steady erosion of the perception that ‘we're all in this together’. The public often felt (with good reason) blamed for failing to adhere to restrictions that were sometimes both byzantine in their complexity and ambiguous in their meaning, which even ministers clearly did not understand, and which were constantly changing. There was often little transparency about the advice the government was receiving—itself an extension of a style of governance that developed during the Brexit era—and at times ministers themselves made claims, for example about progress with testing, the evidence base for policies, or the development of approved vaccines, that were demonstrably untrue. It is scarcely surprising that the integrity and independence of the scientific advice was sometimes questioned too.

The trust sacrificed by the personal and public failures of policy and policymakers also made it harder for the government and scientists to counter a deluge of misinformation coming from parts of the mainstream media as well as from social media. That situation was further complicated by the fact that some of those media outlets were the very ones the government had considered its allies, and sometimes ones with which government members themselves had close ties. (Dominic Cummings alleged that Johnson considered the *Daily Telegraph*, for which he wrote well-paid columns and which has been a prominent source of skepticism about the value of lockdowns, to be his ‘real boss’.)

If, then, there is one prominent lesson to be learnt from the British pandemic experience about how scientific advice feeds into political decision-making, it is about the value of independence and transparency of that advice. During the BSE pandemic of the early 1990s, the Agriculture Minister John Gummer notoriously claimed that all British beef was safe and tried (without success) to persuade his daughter to demonstrate as much by eating a beefburger. As subsequent events showed, this claim was false: several people subsequently died from Creutzfeld–Jacob disease caught from contaminated meat. The Phillips Report (see endnote 1) in the wake of that episode stressed that it is vital the public be able to see the science on which government statements and policies are based. It stated that:

— To establish credibility it is necessary to generate trust.
— Trust can only be generated by openness.
— Openness requires recognition of uncertainty, where it exists.
— The importance of precautionary measures should not be played down on the grounds that the risk is unproved.
— The public should be trusted to respond rationally to openness.
— Scientific investigation of risk should be open and transparent.
— The advice and the reasoning of advisory committees should be made public.
— The trust that the public has in Chief Medical Officers is precious and should not be put at risk.

Arguably each of these tenets was disregarded during the COVID-19 pandemic.

Beyond these considerations, the pandemic has made it clear how contingent the effectiveness of scientific and technological interventions are on wider social factors, in particular socioeconomic inequalities and poor public health [25]. No recommendations for lockdowns or restrictions can be fully effective if people cannot afford to adhere to them. Widespread testing makes little difference unless the results can be conveyed quickly and then acted on. Deadly infectious diseases will expose and exploit the inequalities in society: the greatest vulnerability to infection and serious illness tend to occur in areas of greatest deprivation, making social inequality not only iniquitous in itself but also a public-health hazard—not least because of the way it exacerbates risk factors such as obesity and chronic respiratory problems [26]. And outcomes depend on clear and honest communication, and an ability to overcome agents of misinformation—an issue initially for compliance with restrictions, and now for the vaccine uptake essential to fully suppress the virus.

The message, then, is one that no serious scholars of science, society and history would ever have doubted: science does not operate in a social or political vacuum, but is shaped by as well as shaping the societies and cultures in which it unfolds. Scientists must recognize that offering objective advice to policy makers and the public is not the sole extent of their responsibilities, and that facts may not be self-evident and ineluctable or have obvious or inevitable implications. Politicians, meanwhile, should not use science as a shield against making (or accepting responsibility for) difficult decisions, and should acknowledge that scientific advice is likely to be more effective when it is genuinely independent, autonomous and transparent. We cannot expect good public health to be valued and nurtured if political health is poor.

## Endnotes

1 https://webarchive.nationalarchives.gov.uk/20060525120000/http://www.bseinquiry.gov.uk/report/index.htm.

2 https://www.ons.gov.uk/peoplepopulationandcommunity/birthsdeathsandmarriages/deaths/bulletins/deathsinvolvingcovid19bylocalareasanddeprivation/deathsoccurringbetween1marchand30june2020.

3 https://www.ons.gov.uk/peoplepopulationandcommunity/birthsdeathsandmarriages/deaths/articles/coronavirusrelateddeathsbyethnicgroupenglandandwales/2march2020to10april2020.

4 https://www.gavi.org/covax-facility.

5 https://www.who.int/news/item/23-09-2020-managing-the-covid-19-infodemic-promoting-healthy-behaviours-and-mitigating-the-harm-from-misinformation-and-disinformation.

## Data accessibility

This article has no additional data.

## References

[1] MacMillan M. 2000 The world after COVID: a perspective from history. In COVID-19 and world order (eds H Brands, FJ Gavin), pp. 40–55. Baltimore, MD: Johns Hopkins University Press.

[2] Wise J. 2021 COVID-19: highest death rates seen in countries with most overweight populations. Br. Med. J. **372**, n623. (10.1136/bmj.n623) 33664055

[3] Vaughan A. 2020 Why have there been so many coronavirus deaths in the UK? *New Scientist* 6 June 2020.

[4] Hughes L. 2020 Earlier lockdown could have halved UK deaths, says PM's ex-adviser. *Financial Times* 10 June 2020. See https://www.ft.com/content/820761d1-8bb2-4386-9637-ce4280d7f6e3

[5] Wolf M. 2020 What the world can learn from the Covid-19 pandemic. *Financial Times* 24 November 2020. See https://www.ft.com/content/7fb55fa2-4aea-41a0-b4ea-ad1a51cb415f

[6] Gans J. 2020 Economics in the age of COVID-19. Cambridge, MA: MIT Press.

[7] WHO. Transmission of SARS-CoV-2: implications for infection prevention precautions. World Health Organization Scientific Brief, 9 July 2020. See https://www.who.int/news-room/commentaries/detail/transmission-of-sars-cov-2-implications-for-infection-prevention-precautions.

[8] Lewis D. 2020 Coronavirus in the air. Nature **583**, 510-513. (10.1038/d41586-020-02058-1) 32647382

[9] Jee C. 2020 The CDC has finally acknowledged that the coronavirus can be airborne. *MT Technology Review* 6 October 2020. See https://www.technologyreview.com/2020/10/06/1009424/the-cdc-has-finally-acknowledged-that-the-coronavirus-can-be-airborne/.

[10] Brienen NC, Timen A, Wallinga J, Van Steenbergen JE, Teunis PF. 2010 The effect of mask use on the spread of influenza during a pandemic. Risk Anal. **30**, 1210-1218. (10.1111/j.1539-6924.2010.01428.x) 20497389 PMC7169241

[11] Suess T et al. 2012 The role of facemasks and hand hygiene in the prevention of influenza transmission in households: results from a cluster randomised trial; Berlin, Germany, 2009–2011. BMC Infect. Dis. **12**, 26. (10.1186/1471-2334-12-26) 22280120 PMC3285078

[12] Lewis D. 2020 Is the coronavirus airborne? Experts can't agree. Nature **580**, 175. (10.1038/d41586-020-00974-w) 32242113

[13] Baynes C. 2020 Coronavirus: face masks could increase risk of infection, medical chief warns. *The Independent* 12 March 2020. See https://www.independent.co.uk/news/health/coronavirus-news-face-masks-increase-risk-infection-doctor-jenny-harries-a9396811.html.

[14] Hancock M. 2020 We must all do everything in our power to protect lives. *Sunday Telegraph* 14 March 2020. See https://www.telegraph.co.uk/politics/2020/03/14/must-do-everything-power-protect-lives/.

[15] Ferguson NM et al. 2020 Imperial College COVID Response Team Report No. 9. 16 March 2020. See https://www.imperial.ac.uk/media/imperial-college/medicine/sph/ide/gida-fellowships/Imperial-College-COVID19-NPI-modelling-16-03-2020.pdf.

[16] Buss LF et al. 2020 Three-quarters attack rate of SARS-CoV-2 in the Brazilian Amazon during a largely unmitigated epidemic. Science **371**, 288–292. (10.1126/science.abe9728) 33293339 PMC7857406

[17] Sridhar D, Gurdasani D. 2020 Herd immunity by infection is not an option. Science **371**, 230-231. (10.1126/science.abf7921) 33446540

[18] King D. 2020 Being open and transparent about science. FST J. **22**(8), 10-11.

[19] Fancourt D, Steptoe A, Wright L. 2020 The Cummings effect: politics, trust, and behaviours during the COVID-19 pandemic. Lancet **396**, 464-465. (10.1016/S0140-6736(20)31690-1) 32771083 PMC7613216

[20] Ball P. 2020 The lightning-fast quest for vaccines. Nature **589**, 16-18. (10.1038/d41586-020-03626-1) 33340018

[21] Lieven A. 2020 Climate change and the nation state: the case for nationalism in a warming world. New York, NY: Oxford University Peess.

[22] Bobbitt P. 2000 Future scenarios: "we are all failed states, now". In COVID-19 and world order (eds H Brands, FJ Gavin), pp. 56–71. Baltimore, MD: Johns Hopkins University Press.

[23] Ball P, Maxmen A. 2020 Battling the infodemic. Nature **581**, 371-374. (10.1038/d41586-020-01452-z) 32461658

[24] Birch J. 2021 Science and policy in extremis: the UK's initial response to COVID-19. Eur. J. Phil. Sci. **11**, 90. (10.1007/s13194-021-00407-z) PMC8385263 34457091

[25] Raleigh VS. 2020 UK's record on pandemic deaths. Br. Med. J. **370**, m3348. (10.1136/bmj.m3348) 32887679

[26] Green D, Filkin G, Woods T. 2021 Our unhealthy nation. Lancet Healthy Longev. **2**, E8-E9. (10.1016/S2666-7568(20)30062-3) PMC9529172 36098161

